# The significance of Q-methodology as an innovative method for the investigation of affective variables in second language acquisition

**DOI:** 10.3389/fpsyg.2022.995660

**Published:** 2022-08-18

**Authors:** Xiaodong Li

**Affiliations:** School of Foreign Studies, University of Science and Technology Beijing, Beijing, China

**Keywords:** affective variables, complex dynamic systems theory (CDST), Q-methodology, second language acquisition (SLA), affective factors

## Abstract

Q methodology has been used in a variety of fields to employ a scientific approach to dealing with subjectivity; yet, its use has just gained momentum in the second language acquisition (SLA) domain recently (Damio, 2016). The present paper argues that Q methodology is remarkably efficient in representing the dynamic quality of complex systems involved in the language learning process, which is, thus, compatible with the complexity and dynamic systems theory (CDST). As Q methodology enjoys advantages of both qualitative and quantitative lines of research (Irie, 2014), it helps to explore and reflect L2 learners’ subjective views and perceptions about their emotions in an L2 class in a comprehensive manner. With the current growing attention to individuals’ emotional experiences in recent years, SLA research domain is ripe for many scientific inquiries about L2 learners’ affective variables benefiting from this method. The few existing studies in the L2 domain have had interesting findings, which show the Q methodology should be more extensively used in the field to reveal facts about how learners feel in class from a within-individual point of view. Q methodology can hopefully be capable of representing the dynamicity and complexity of the affective variables language learners experience in the interactive network of classroom learning. Thus, it is expected that innovative research methods such as the Q methodology be employed significantly more than before in the dynamic phase of SLA research in the upcoming years.

## Introduction to Q-methodology

Q methodology, a robust approach to the investigation of individuals’ perspectives was first developed in the psychology of education by a British psychologist and physicist William Stephenson in the 1930s (Damio, 2016). This methodology has been applied in different fields of study related to social sciences such as education, psychology, as well as policy-making research, with the advantage of its systematic quality which guarantees comparability and consistency among various studies, and clarity of explanations to the audience (Irie, 2014). Q methodology has been used until now for different research purposes including the investigation of the teachers’ perceptions (Rimm-Kaufman et al., 2006). In the second language acquisition (SLA) domain, its use has been restricted, so far, and the number of studies is limited; yet, there seems to be a rise in the growing signs of interest in Q methodology recently (Irie, 2014; Irie and Ryan, 2014; Collins and Angelova, 2015). Q methodology is an opposing reaction to the prevalence of multivariate factor analysis in the body of research on psychological phenomena (Brown, 1980). Multivariate factor analysis approaches individual behavior from an outside observer’s viewpoint. However, Q methodology approaches human behavior from an inner viewpoint residing within the individual’s self and from his/her eyes (Brown, 1980), and relies on social classification.

In applied linguistics research, Q methodology is considered as a special type of the ordinary Likert-scale survey, which is commonly used in quantitative research (Bartels, 2005). Recent years witnessed several researchers’ beginning to appreciate certain capabilities of the Q methodology (Pemberton and Cooker, 2012; Rodriguez and Shepard, 2013; Irie and Ryan, 2014) to delve into individual language learners’ perspectives. Subsumed under the SLA domain is the individual differences research, with the aim of excavating the overall rules dominating the human mentality and exploring the idiosyncrasies of an individual’s mind (Dörnyei, 2005) which seems to draw on Q methodology. The line of individual differences inquiry looks down on the conventional investigations seeking to present universal models and all-inclusive interpretations of language learners’ attitudes, emotions, and behaviors. Rather, it seeks to reveal more nuances of individual differences in the authentic and natural learning environments (e.g., classroom learning).

The most common application of the Q methodology has been to investigate multifaceted and socially constructed concepts as experienced or expressed by the participants from their own perspectives (Stainton Rogers, 1995; Watts and Stenner, 2005). The existing published Q-method papers about language learning and teaching domain are limited in number (e.g., Irie, 2014; Irie and Ryan, 2014; Kruk et al., 2021). They will be reviewed in the present study. The reason is that Q methodology is still a newcomer to the applied linguistics domain. Though it is considered an original research methodology in SLA, it is capable of presenting a comprehensive cognitive and emotional conceptualization of a given context by associating the dominant emotions, perceptions and speculations of individuals about a particular subject marked by complexity and dynamicity (Irie, 2014). Affective variables involved in a learning environment, subsumed under the psychology of education, are dynamic, complex, and socially created emotional conditions; therefore, Q-methodology can be an appropriate alternative research method to efficiently investigate L2 affective factors. Q methodology, instead of drawing up on a passive response dimension, plays the role of a dynamic instrument to comprehensively represent subjectivity (Watts and Stenner, 2005). It approaches a given context from a subjective point of view and not an objective view, more basically from a context-specific and interpretive perspective and not a positivist one (Jodaei et al., 2021).

It should be mentioned that that the acknowledgment of the Q methodology has been in tandem with the dynamic turn in the field of SLA. Due to the dynamic turn see Dörnyei and Ryan (2015) under the influence of complex dynamic systems theory (CDST), in this field, a shift from variable centered approaches to L2 affective factors with an etic perspective to person-centered approaches to these variables with a subjective emic perspective took place. CDST as a meta-theory holds a context-bound and individualized perspective to psychological constructs in the field of SLA. This means that in a given setting, individual learners might have different affective experiences and this needs to be reflected in the methods used to trace these inter-individual differences in the L2 affective domain.

Another reason for the recent acknowledgment of the Q methodology in the L2 affective domain has been its adaptation to the ergodicity issue in the investigation of individual differences in the L2 affective domain. Since language learners are not ergodic ensembles, individual language learners’ affective experiences are not necessarily what the average experiences (Lowie and Verspoor, 2019). Thus, Q methodology with an inner subjective perspective can provide us with deeper insights into individual differences with respect to L2 affective variables compared to variable centered approaches. In fact, the integration of the dynamic turn and the issue of ergodicity regarding the investigation of L2 affective variables set the stage for the attention to the salience of individual subjective perspective in the L2 affective domain and the application of person-centered approaches such as Q methodology to follow this individual orientation.

## Introduction to L2 affective variables

During the language learning experience, teacher’s and students’ affective factors are to a great extent involved. In fact, the language learning process is approached as a dynamic process that involves both emotional and psychological aspects marked by constant variations within and among learners (MacIntyre and Gregersen, 2012). L2 learners may be unaware of the effect of affective factors such as anxiety, boredom, and enjoyment on their language learning experience. Yet, they are constantly influenced by the dynamicity of these affective variables (MacIntyre and Gregersen, 2012). Language learners’ emotions significantly affect the individuals’ current emotional states (Mates and Joaquin, 2013; Derakhshan et al., 2022). Positive emotions are able to add stability to intrapersonal and interpersonal resources (Fredrickson, 2003; Xie and Derakhshan, 2021), yet negative emotions can limit language learners’ concentration on tasks or even performance (Derakhshan et al., 2021; Pawlak et al., 2021). Positive and negative emotions both show particular behavioral patterns in the learning process which is primarily formed according to a strength of internal or external forces (Wang et al., 2021). L2 learners may begin their experience of language learning at a particular initial level of emotions or affective variables (Larsen-Freeman and Cameron, 2008). Yet, these initial conditions will by no means remain the same, and can change under the influence of the external and internal factors. For instance, the peer’s or teacher’s responses or feedback to the students can result in distinctive emergent forms of emotional reactions shown by language learners.

It is also noteworthy that even small changes in students’ affective states and emotions can have a butterfly effect and seemingly small changes can introduce a tremendous change to the whole network of classroom events (Gregersen et al., 2014). Reviewing the majority of studies on language learners’ affective variables shows that they mostly adopted a trait-oriented approach to the examination of these factors. Quantitative Likert-scale questionnaire surveys fail to reflect the ever-present unanticipated variation in the learners’ affective variables (Mates and Joaquin, 2013). Therefore, innovative research methods, preferably with the characteristics of both qualitative and quantitative research, are needed to examine the dynamics of L2 learners’ affective states (de Bot et al., 2007; Larsen-Freeman and Cameron, 2008; MacIntyre and Gregersen, 2012). According to Larsen-Freeman’s (2016) contention (2008, 2016) of the multi-layered procedures underlying language learning, it can be conceived that these processes are hardly linear and, rather, seem to be emergent differently in different language learners across different points or periods of time. Therefore, exploring language learners’ affective variables needs to change from a state-oriented view to a more realistic dynamic view (Gregersen et al., 2014). Thus, it seems reasonable that Q method, with qualities of both quantitative and qualitative research (Irie, 2014), be fit for the investigation of L2 affective variables. Below, the suggested procedure will be delineated.

## The procedure of Q-method to study L2 affective factors

Regarding the origin of the method, the Q methodology questioned the assumptions of factorability of the viewpoints in terms of the items of a questionnaire because such items are mainly developed based on an outsider perspective which do not necessarily reflect the individuals’ subjective perspective see Watts and Stenner (2005). The Q methodology regarded the subjective view points of individuals as the sources of factorability of viewpoints. The number of participants, in the related literature on Q methodology, is ordinarily specified to be between a minimum number (i.e., one third of the included items) (Drakeva, 2017) and a maximum which means the number of the whole items included (Slaughter et al., 2019). Q method does not require a great number of subjects due to its exploratory and qualitative nature. Its goal is not to generalize the results, but instead it aims to point to the significance of each perspective for the participating subjects (Slaughter et al., 2019). Concerning the representativeness of the sample to be selected from among the research population, Brown (1980) contends that with a collection of items of the same scope around the topic of research, similar factors could be extracted from other participants and, therefore, factors can be considered as generalizable. Similarly, Thomas and Baas (1992) argued that, as a few attitudes prevail on a given matter, apparently similar yet divergent Q sorts submitted to various subjects can help to reveal the factors that underlie the same attitudes. The data collecting and analyzing procedures are five to be included in Q methodology. These include: formulating some research question(s), constructing a Q set, disseminating the Q sorts, conducting the statistical procedures, and explaining the findings.

### Stage 1: formulating a research question

Putting forth a suitable research question is of an utmost importance in a Q method work of research. The content and holistic design of a Q set depend on the quality of research question. Due to the great implications of exploring L2 affective factors, some Q method research may be developed to explore different common experiences of L2 learners concerning the view of conditions that elicit a specific affective factor (e.g., boredom, enjoyment, and anxiety) in the L2 learning setting. The research question can be framed as below. In the development of a Q set and the items of a scale of an L2 affective variable, the literature review or some pilot interviews are referred to as the main sources but when it comes to the Q set, larger number of statements are considered in the Q set because it is supposed to provide feasible extraction of subjective viewpoints in the Q sorting stage.

*Research question frame*: What are the different forms of individual [affective variable]-laden states in the context of L2 learning?

### Stage 2: constructing a Q set

In Q methodology, a Q set helps to collect the relevant data. It is comprised of several diversified statements which aim to elicit the respondents’ attitudes toward a specific topic. In a study using the Q method, for a Q set, the included statements are derived from the existent literature about the affective factors of interest and the measurement questionnaire surveys that already existed and were used in former works of research. This stage aims to cover as much as possible the related discourse around the affective variable of interest. A specific challenge learners might face in the application of the method in the L2 affective domain might be related to the development of the construction of a Q set. Mastery of the literature review of an affective variable and the type of Q sorting adopted in conducting a Q methodology are among the challenges learners might face in the application of the method.

### Stage 3: disseminating the Q sorts

**FIGURE 1:**
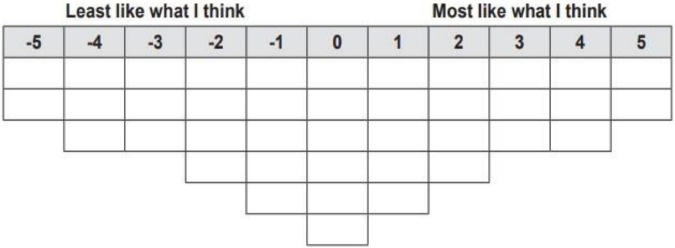
A sample Q grid (adapted from Damio, 2016).

The most important part of a Q work of research is the act of collecting data in a sorting phase. Therefore, initially, the subjects should sort the statements about the affective variable to be investigated, from the least determining (e.g., −5) to the most determining (e.g., +5) reasons for experiencing that particular affective variable in an L2 class. In a Q study, the respondents are required to sort the included statements according to a pre-determined pattern guided by a sorting grid (see Figure 1), which is a pre-specified pattern of distribution. This pre-specific distribution is a part of a Q method work of research which allows respondents to arrange the statements according to a pre-set pattern. Even when a pattern is not instantly attractive to the respondent, the act of organizing his/her thoughts to the pre-set pattern makes him/her carefully ponder up on the potential relationship(s) between and among the statements, and not to merely consider each item in isolation.

### Stage 4: doing statistical analysis

The statistical procedure of a Q method entails an inverted factor analysis. PQ Method (Schmolck, 2002) is used in the analytic procedure to provide the researchers with the primary by-person correlation matrix. In the analytic procedure, the classification of the factors is done according to the rank-ordering of all the statements in comparison to one another, and not the commonality of independent answers to every statement, which commonly takes place in Likert scale type questionnaire surveys (Dörnyei et al., 2015). Subsequently to the extraction of factor, varimax rotation, and a manual adjustment, the findings are interpreted based on the factor arrays. The last stage is followed using the table of factor ranges and configuring the items. The major point in the analytic procedure is the highest and lowest scores obtained from the factor loading.

### Stage 5: explaining the results

The findings obtained from the analysis of the other items within the Q set, demographic variables, and the additional possible methods of data collection (e.g., interview) are used to state the conclusions. A narrative style may be employed for reporting the interpretation of factors. This style entails the ordering of the associated items of particular factors and linking them to each other for generating a single and solid description of the viewpoint to the factors (Watts and Stenner, 2012). In addition, the interview data obtained from the typical subjects could be employed to form a comprehensible perception of the respondents’ subjective accounts. The factor analysis can follow to extract several primary factors.

## Exemplary Q-methodology studies in second language acquisition

Irie (2014) introduced Q method as a way of eliciting the subjective evaluations of complex processes. This researcher pointed out the prevalence of the method in many fields of social sciences and the scarcity of its use in SLA research. Irie (2014) perceived the peculiarity of Q-method in that it enjoyed characteristics of both qualitative and quantitative approaches to research. In this study, Irie elaborated on the Q method and proved how it could benefit the emergent needs of the SLA domain of research. Irie (2014) discussed how the Q-method could be applied in SLA research and specifically focused on the parallel growth of this method in the 1930s and the present criticisms of the conventional mentalistic approach to SLA research. This researcher also cited another earlier study by Irie and Ryan (2014) to explain how the emphasis on the integrative view of subjectivity is translated in a procedural plan. Recommendations were finally made for applying Q method in different areas of L2 teaching and learning.

Damio (2016) studied autonomy in language learning using the Q method. This researcher aimed to exemplify a Q method applied in SLA studies, and managed to extract four factors related to the variable of interest from students’ accounts.

Irie et al. (2018) used the Q method to explore pre-service English language teachers’ mindset in an Austrian university. This study explored teachers’ perceptions of their own teaching efficiency and also aimed to contribute to the methodological collections of research methods in SLA domain. They found that the most prevalent mindset in the pre-service teachers is related to a firm belief in the learnability of the more technical dimensions of instructions, whereas communicative skills showed to be considered more as a natural talent instantiated in an individual. A pedagogical implication of this study was that teacher training courses should more seriously consider the direct investment on the instructions’ interpersonally communicative load. Also, Irie et al. (2018) reported that teacher mindset grew by the individuals managing different sets of background theories, who did not tend to adhere to the conventional dichotomous conceptualization of mindset.

Lundberg (2019) used the Q method to investigate teachers’ perceptions of multilingualism and multilingual students in elementary schools in Sweden. This researcher used the Q method to combine qualitative and quantitative data analytic procedures. The Q material used in this research gave the respondents the language they needed to express their evaluative accounts. Two Q sets of items were used, one exploring the attitude toward the variable of interest and the other was about the recommended pedagogical reactions to the present state of multilingualism. Different sources of data were used to construct the items. Using inverted factor analysis as well as an abductive interpretation, a total number of three sets of teachers’ beliefs were extracted. The descriptions reflected the teacher participants’ complex perception of the classrooms marked by multilingualism. The findings showed that, on average, teachers’ perception of multilingualism was positive. So was their attitude toward multilingual learners. The teachers allowed for trans-languaging in class. Yet, negative views were also present about monolingual ideologies and could be expected to cause problems for executing pluralistic rules and regulations in the academic environment. Overall, this research contributed to the heated discussion about the advantages of the present state of multilingualism in academic settings.

Alkhateeb et al. (2020) used the Q method to show how Qatar University’s local and foreign stakeholders viewed the prospects of the language policy of university in the curriculum. These researchers used the Q method to discover social attitudes to three academic language policy alternatives, suggested to the higher administration of the university, by a technical organizing team, to meet the strong language-related needs. The findings concerning the essentiality of integrating the foreign language course in the curriculum were raised to handle the association of English and Arabic together in the academic program.

Jodaei et al. (2021) explored the mutual effect of English language teacher and student motivation in Iran. They used the Q methodology to ensure the systematicity and subjectivity of the findings. They also interviewed the most typical individuals from each factorial group to investigate the students’ points of view about the interrelationship between teacher and learner motivation. They used a hybrid type of Q sampling to construct tens of items about the association of learner and teacher motivation. They used the *PQ Method*, an inclusive statistical package for Q method to intercorrelate the Q sorts and conduct a factor analysis. They finally extracted four factors. To identify and interpret the different extracted descriptions of second language motivation, component arrays and qualitative procedures were used. The extracted components showed that the students had distinctive typical attitudes toward the interrelationship between learner and teacher motivation.

Kruk et al. (2021) used a Q method to explore individual learners’ subjective experience of boredom in an L2 class. They explored Iranian English language learners’ perceived causes of boredom in class. A hybrid-type Q sampling was used to this purpose, which led to the production of 40 statements about the causes of boredom. PQ Method was used to do the statistical analyses. The Q sorts were correlated with each other and a factor analysis followed. Three factors were derived and rotated. These included teacher-caused boredom, learner-caused boredom, and activity-caused boredom. This research also revealed that different typical students experienced boredom differently.

Vanbuel (2021) explored the extent to which different stakeholders viewed the foreign language learning policy effective. This researcher used the Q-methodology to delve into the unexpressed attitudes of stakeholders toward the language learning rule in Belgium. Different stakeholders working in different sections of the policymaking process in the academic setting sorted more than 50 statements about the variable of interest. They were also interviewed. The results led to the extraction of four distinctive perspectives. This research added further evidence for the fact that the whole stakeholders engaged in the implementing phase, reformed and adapted formal rules and regulations to their personal level of understanding. This study showed how different stakeholders perceived the policy in their own way. The findings allowed for determining how future rules and regulations can satisfy the local requirements.

Employed the Q method to investigate English language learners’ subjective accounts of foreign language enjoyment for a whole duration of an online L2 course during the COVID-19 pandemic. Their analysis revealed three different categories of subjective accounts. The first group focused mainly on teachers’ qualities and performance, while the other two groups relied more on student autonomy and social activity. This researcher also reported that the derivation of these factors was at the cost of neglecting others. For instance, interactions among peers were ignored because the classes were inclined toward autonomous learning.

## Prospects of Q methodology for investigating L2 affective variables

As the review of SLA studies using Q methodology showed, the literature is still limited in size, and the existing body of research has emerged only out of the past decade. It is evident that the use of Q method has been only recently appreciated in applied linguistics, and still has a long way ahead. More limited is the number of studies that used the Q method to explore L2-related variables. The number is smaller than a handful (e.g., Jodaei et al., 2021; Kruk et al., 2021). Though limited in number, the findings of these studies have been rich and illuminating for the CDST-led line of inquiry in SLA. Q-method, with its unique advantages, is capable of representing the complex development of learners’ or teachers’ emotions within the context of classroom learning. Thus, it is expected to be embraced more in near future in SLA studies. I already elaborated on the procedure of Q method when it is to be applied for exploring L2 affective variables. Thus, this procedure can be effectively used to formulate relevant research questions followed by rigorous research designs. Since the method is quite novel in both the field of SLA and the L2 affective domain, some differences might be seen in the studies using this method in the L2 affective domain see Kruk et al. (2021). These differences are related to the development of the Q sets and Q sorting due to the specific literature of each L2 affective variable.

Q methodology has the power of eliciting L2 learners’ subjective evaluations. An in-depth understanding of how language learners or teacher feel during the emotion-laden language learning experience has some implications for the pedagogical context. The implication of this study for those involved somehow in the language teaching or learning experience is recognition of the presence of a variety of learner prototypes concerning how they experience a certain affective variable (e.g., motivation, anxiety, and enjoyment) in the language learning process. For example, being aware that every prototypic learner is motivated in a certain way or gets bored in class for a certain reason helps teachers or curriculum designers to better and more appropriately design the session content and the tasks and activities included to preclude negative emotions and feed into the positive ones. Teachers can be better prepared to see different patterns of, for example, enjoyment or boredom in class from different students, and do not use the same strategies for all students. Also, given the subjective exploratory nature of the Q methodology, the recent two studies on boredom and enjoyment in the L2 affective domain (Kruk et al., 2021) have contributed to the literature of these two constructs by providing a systematic exploration of the factors underpinning these two constructs from a subjective inner perspective. However, still many language learning emotions are left unexplored through the Q method (e.g., anxiety, passion for learning, and stress), which can be explored in depth, and have much to contribute to the dynamic phase of SLA research.

## Conclusion

Q methodology can help to illuminate and deepen our horizons to certain dimensions of SLA which have been largely explored so far through the conventional mentalistic perspectives (Irie, 2014). Q method helps to remove the obstacles between human mentality and emotions and also the commonplace categorizations in order to overthrow the kind of black and white or 0–1 way of thinking (Ortega, 2010). The Q methodology described in the present study and its procedures, particularly the act of Q sorting, motivates the individuals to state their subjective judgment about the sources of a certain affective variable they experience in L2 learning classes. As pinpointed by Irie (2014), an idiosyncratic and unique feature of the Q method is that it ensures the individual respondents’ active participation in the study procedure, in the data collection phase. Sorting the items about a particular emotion involved in the L2 learning experience actually gives the student participant precious chances to think carefully about their own feelings and emotions (Cooker and Nix, 2011), and such an ecological aspect encouraged Pemberton and Cooker (2012) to suggest that Q method may be also employed as an instructional task to increase students’ self-awareness and independence.

The commonly used instruments included in multivariate factor analysis or R methodologies (opposite to the Q methodology) fail to conjure up a comprehensive and representative image of the individuals’ points of view. Therefore, the researchers who hope to employ the Q method are recommended to clearly explain all the stages involved one by one to the respondents. Watts and Stenner (2005) cautioned against highly prevalent misinterpretations of the Q pattern analysis and also Q sorting. Despite the fact that there are a wide range of research methods to delve into affective variables in a second language learning context, Q methodology is maintained to be truly fruitful as it comprehensively embraces the dynamicity of complex systems in L2 learning. Since Q method enjoys the qualities of both qualitative and quantitative research (Irie, 2014), it is capable of paving the way for an in-depth exploration and representation of L2 learners’ subjective evaluations and assessments completely. SLA research domain with its present-state growing attention to language learners’ and teachers’ affective states is ripe for further research benefiting from Q methodology.

## Suggestions for further research

Q methodology is considered a truly original method of research which is capable of portraying a cognitive and affective image of a specific setting by associating the major feelings and conceptualizations of people about a complex phenomenon. Q methodology can do it with no need for polarizing the expressed evaluation of each individual into categories which are fixed in advance. Q methodology is a beneficial alternative technique for inquiry, sensitive to the needs of the ever-burgeoning theoretical viewpoints and dynamicity of the variables in the SLA research. Due to the ergodicity issue in the investigation of L2 affective variables, the classic nomothetic or variable centered approaches to the investigation of L2 affective variables have been replaced or accompanied with idiographic or nomothetic approaches like Q methodology see Gkonou et al. (2017). Thus, the application of these emerging methods does not underestimate the contribution of variable centered approaches to our understanding of L2 affective variables. Instead, it aims to uncover new dimensions of L2 affective variables which have not been explored yet.

Despite the alleged usefulness of the method to the exploration of affective variables in SLA domain, the existing literature is still significantly limited in number. Yet, the quality of findings and their pedagogical implications is so high that it is strongly suggested to use this method more than ever before in the field. L2 related affective variables, due to their developmental nature lend themselves very well to in-depth exploration of individual participants’ subjective views. Among the affective variables that have been so far explored *via* the Q method are foreign language enjoyment, boredom, motivation, and immunity. Still, many are left untouched, such as anxiety, passion for learning, stress, self-esteem, and so on. These are all welcomed in the CDST-directed line of research in applied linguistics, which shows interest in investigating complex dynamic systems. Q method can be used to unravel different causes of negative affects (e.g., boredom, anxiety, and stress) as perceived by language teachers or students, who are all directly involved in the language learning experience in the immediate context of classroom learning. The pedagogical implications will be incomparably valuable, as they can help teachers to be better equipped to deal with different prototypical language learners experiencing a certain emotion in a certain way in class. The findings of studies using the Q method to explore L2 affective variables can be integrated within the teacher training courses for preservice L2 teachers.

## Author contributions

The author confirms being the sole contributor of this work and has approved it for publication.
